# Automated Visual Inspection for Precise Defect Detection and Classification in CBN Inserts

**DOI:** 10.3390/s24237824

**Published:** 2024-12-07

**Authors:** Li Zeng, Feng Wan, Baiyun Zhang, Xu Zhu

**Affiliations:** 1 School of Mechanical and Electrical Engineering, Zhejiang Industry Polytechnic College, Shaoxing 312000, China; 20080005@zjipc.edu.cn; 2 Ningbo Jiapeng Machinery Equipment Manufacturing Co., Ltd., Ningbo 315101, China; mark@cn-jiapeng.com; 3 Ningbo Institute of Dalian University of Technology, Ningbo 315032, China; 4 State Key Laboratory of High-Performance Precision Manufacturing, Dalian University of Technology, Dalian 116024, China

**Keywords:** CBN insert, defect detection, visual inspection technology, deep learning

## Abstract

In the high-stakes domain of precision manufacturing, Cubic Boron Nitride (CBN) inserts are pivotal for their hardness and durability. However, post-production surface defects on these inserts can compromise product integrity and performance. This paper proposes an automated detection and classification system using machine vision to scrutinize these surface defects. By integrating an optical bracket, a high-resolution industrial camera, precise lighting, and an advanced development board, the system employs digital image processing to ascertain and categorize imperfections on CBN inserts. The methodology initiates with a high-definition image capture by the imaging platform, tailored for CBN insert inspection. A suite of defect detection algorithms undergoes comparative analysis to discern their efficacy, emphasizing the impact of algorithm parameters and dataset diversity on detection precision. The most effective algorithm is then encapsulated into a versatile application, ensuring compatibility with various operating systems. Empirical verification of the system shows that the detection accuracy of multiple defect types exceeds 90%, and the tooth surface recognition efficiency significantly reaches three frames per second, with the front and side cutting surfaces of the tool in each frame. This breakthrough indicates a scalable, reliable solution for automatically detecting and classifying surface defects on CBN inserts, paving the way for enhanced quality control in automated, high-speed production lines.

## 1. Introduction

Cubic boron nitride (CBN) inserts are integral to modern industrial production [1,2,3], widely employed in diverse sectors such as machine tool processing [4,5,6,7], mold manufacturing [8,9,10], and automotive parts fabrication [11,12,13,14]. Despite their significance, these inserts are susceptible to various manufacturing defects like cracks, wear, and fractures due to factors related to production processes, material quality, and operational environments. Such imperfections can substantially diminish the durability and precision of the tools, with severe cases posing potential safety risks [15]. Consequently, the accurate and prompt identification of defects in CBN inserts is of critical importance to maintaining high standards of quality and safety in industrial operations [16]. While production lines for CBN inserts manufacturing have achieved a degree of automation, significant reliance on human intervention persists in the realm of quality assurance. Manual inspection methods continue to consume upwards of 40% of the total manufacturing time for individual components [17]. Human participation in inspection tasks stands as a considerable bottleneck, impeding the productivity of the automated production line. An automated, agile and cost-effective inspection solution can assist in maximizing the benefits of digitization and product quality monitoring within the production chain.

As Industry 4.0 reshapes manufacturing, machine vision inspection tech has taken center stage for its pivotal role in automating quality control and monitoring. It empowers machines to observe physical activities and, following this, to perform extraction, processing, and analysis critical for informed decision-making. The fusion of cutting-edge deep learning algorithms has been a game changer, driving the creation of sophisticated vision-based solutions. These advancements are not just ramping up accuracy—they are slashing costs, marking a revolutionary shift in how industries ensure the caliber of their products. The image-based target detection model serves as the core of machine vision detection technology [18]. which is a commonly employed approach that can automatically identify targets within images, offering notable advantages in efficiency, accuracy, and stability.

Presently, YOLO [19] (i.e., You Only Look Once) has emerged as a focal point in the realm of target detection research, renowned for its rapid processing speed and high level of accuracy, garnering widespread recognition. Bharat Mahaur et al. [20] introduced architectural changes to the YOLOv5 model to improve its performance in the detection of small objects without sacrificing the detection accuracy of large objects. It is capable of boosting the detection accuracy and speed without greatly increasing the model complexity. Wuzhen Huang et al. [21] proposed a detection system to detect surface defects on the ground cell phone ceramics backplane based on the YOLOv3-tiny model. The experimental results show that the recognition accuracy of the model can reach 89.9%, and the model on Android can achieve two frames per second (FPS). Nevertheless, the detection accuracy remains suboptimal, and manually labelling thousands of images presents a significant workload. Ambadekar et al. [22] used a microscope to collect 1183 images of the back surface of turning tools with different wear degrees, divided the wear degrees into three levels: low, medium and high, and used CNN to train the classification model, so as to achieve the prediction of tool wear state. You Zhang et al. [23] used convolutional neural networks to locate and extract the wear area of milling cutters and then used the least square method to calculate the wear amount of cutting tools. Tongjia Zhang et al. [24]. improved the Yolov3-tiny model to extract the edge of the milling tool blade and then used traditional image processing methods to detect and evaluate defects. This approach enhances defect detection accuracy compared to relying solely on deep learning. Furthermore, it exhibits superior robustness to variations in lighting conditions compared to traditional image processing methods. Xuefeng Wu et al. [25] established an on-machine detection system on a CNC milling machine by obtaining all the tool wear images in the processing gap through an industrial camera and establishing an image dataset. Experimental findings indicate that this model achieves an average recognition accuracy of 96.20%. Simultaneously, an enhanced automatic detection algorithm for tool wear values is introduced in conjunction with the identified tool wear types. Nevertheless, its automatic detection device is relatively simplistic, capable of detecting only one tool surface at a time. Moreover, the proposed wear detection algorithm exhibits low efficiency and limited generalization ability. Thomas Bergs et al. [26] successfully classified color images of ball end mills, end mills, inserts, and drills through a self-trained CNN model, with a test accuracy of 95.6%. Nonetheless, training models from scratch demands a considerable amount of time and labor, as well as stringent criteria for designers. The sphere of advanced manufacturing stands on the precipice of innovation with the advent of machine vision detection technology, particularly in the quality control of cutting tools. Notably, the specialized domain of CBN inserts, revered for their unparalleled hardness next to diamonds, presents unique challenges. Despite the critical importance of flawless surfaces for optimal performance, more research needs to be done focusing on the visual detection of the diverse array of surface defects that can afflict these inserts. In the realm of visual inspection technology for defect classification and extraction, the accuracy of detection algorithms has received substantial attention. However, this focus often needs to be revised to include other critical parameters, such as detection speed, target size, and precision, which are equally crucial in industrial production contexts. Furthermore, traditional algorithms for surface defect detection tend to be constrained to specific products and scenarios, needing more versatility in addressing the complexity of interactions among different defect types and the precise measurement of defects. At the same time, a comparative analysis was conducted against renowned object detection algorithms such as Faster R-CNN [27], SSD [28], and RetinaNet [29].

This study introduces a specialized surface defect detection system designed explicitly for CBN inserts to address these gaps. The system integrates the enhanced YOLOv5s algorithm with an industrial-grade camera to capture detailed images of defect areas on inserts. This approach leverages the computational efficiency and reduced latency of YOLOv5s to provide timely and accurate visual inspections necessary for quality control in manufacturing environments. By establishing a comprehensive data-driven framework, the system is uniquely tailored for the insert’s specifications, enhancing the ability to identify and detect defects at the production level with unprecedented precision and efficiency.

In Section 2, the construction of the overall framework, the image acquisition system and the types of surface defects of CBN tools are introduced. Section 3 and Section 4 respectively introduce the image processing algorithm and the analysis of experimental results. The discussion and summary are explained in Section 5.

## 2. Methodology

To fulfill the rigorous requirements of industrial production, the defect detection system for CBN inserts is engineered to balance detection accuracy with operational speed. This necessitates a system architecture that is intricately designed and optimized to perform defect detection tasks with precision and efficiency.

**Figure 1:**
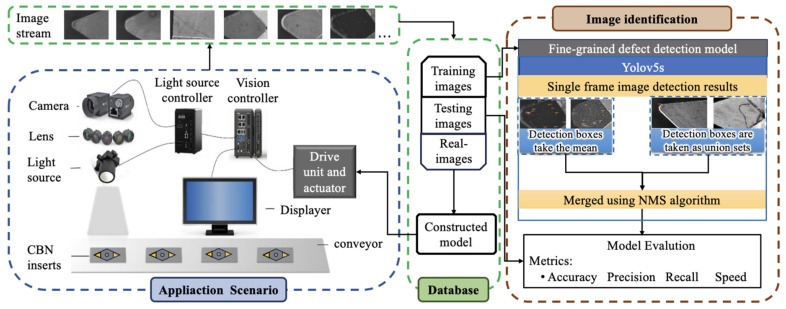
Overview of methodology.

Figure 1 delineates the defect detection methodology for CBN inserts. Prior to image capture, the surfaces of CNC-machined CBN inserts must be meticulously cleaned to remove any oil and debris. Following this preparation, the inserts are positioned on the specially designed inspection platform for analysis. Focused attention is given to the camera setup, where the depth of field is adjusted to capture images of both the rake and flank faces optimally. The images obtained are forwarded to the detection model, which processes and outputs the findings directly onto a display for immediate review. The methodology encompasses three integral components: the establishment of a comprehensive dataset, the generation of a robust training model, and the meticulous implementation of the detection system. This section delves into a detailed exposition of each component, laying out the groundwork for the dataset creation, elucidating the nuances of the deep learning detection model, and detailing the architecture of the defect detection system, all tailored specifically for CBN inserts.

### 2.1. Image Acquisition

**Figure 2:**
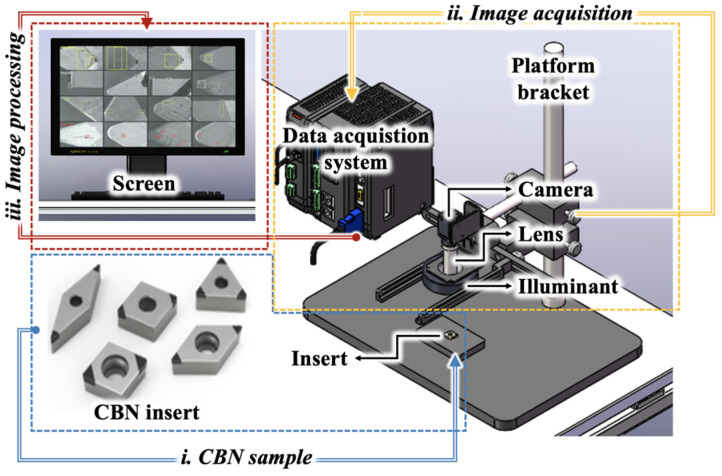
Defect detection and classification system.

Figure 2 presents the schematic diagram of the bespoke experimental platform designed for tool image detection. This platform incorporates several key components: the industrial camera (MV-CS060-10GM, Hikvision, Hangzhou, China), a telecentric lens (MVL-MY-2-65-MP), a precision light source (HL-RD0-90-4-W), a sophisticated visual controller, and a stable platform support. The industrial camera was utilized to obtain high-resolution images of both the flank and rake surfaces of inserts. The telecentric lens principally ensures the collection of insert surface defect images with high fidelity. This lens is characterized by a target surface size of 1/1.8 inches, an optimal object distance of 65 mm, and a depth of field of 0.3 mm. It facilitates a maximum imaging range of 3.69 mm × 2.46 mm, which, in conjunction with the industrial camera’s high resolution of 3072 × 2048 pixels and a pixel size of 2.4 µm × 2.4 µm, guarantees the precision required for accurate defect assessment. Table 1 tabulates other parameters of the components used in the study.

### 2.2. Image Preprocessing

To refine the quality of the training dataset and enhance image definition, this study implements a series of image preprocessing measures on the captured surface images of CBN inserts. The aim is to exclude irrelevant information from the images, thus amplifying the accuracy and effectiveness of the defect detection model. Preprocessing transforms the raw image data into a format more suitable for detailed analysis, preventing the direct application of unrefined image samples in the analysis phase, which could introduce errors in defect identification. The principal image preprocessing techniques utilized in this experiment encompass image stitching to create a comprehensive view of the surface, conversion to grayscale to reduce computational complexity, and application of filtering methods to smoothen and improve the quality of the image data.

The visual inspection platform employed in this study offers the capability to magnify the target tool by 50 to 70 times. The industrial camera that captures images provides a 6-megapixel resolution, producing images of 3072 × 2048 pixels. Due to the industrial camera’s limited field of view, it is not feasible to capture a complete image of the CBN insert’s rake face in a single shot. Therefore, image stitching is essential. In this context, the Scale-Invariant Feature Transform (SIFT) [30] algorithm is employed for its robust feature detection and image alignment capabilities. Utilizing SIFT, feature points are matched, and these correspondences are used to compute the homography matrix with the Random Sample Consensus (RANSAC) algorithm [31]. This matrix, denoted as H, is instrumental in transforming pixel point coordinates from the initial images to their new aligned positions, resulting in a composite image that is the culmination of the stitching process.

In processing color images to grayscale, both linear and nonlinear methods are scrutinized to determine the most effective approach for highlighting defects. A nonlinear transformation, precisely a grayscale stretching technique, is selected for its superior contrast enhancement between defective areas and their surroundings on the CBN inserts. This method distinctly accentuates the surface anomalies. Further clarity is achieved through image smoothing filtering. A comparative analysis of several algorithms, including mean, median, Gaussian filtering, and wavelet transform, is conducted, with performance gauged by indicators such as Mean Squared Error (MSE) and Maximum Absolute Mean (MAM). Median filtering emerges as the optimal choice due to its minimal MSE and MAM values, coupled with its simplicity and effectiveness in noise reduction. However, this process can introduce blurring, which is corrected using the Laplacian operator, sharpening the image and restoring lost detail during median filtering. These preprocessing operations are pivotal for optimizing the quality of training samples, eliminating extraneous data, improving model accuracy, and streamlining subsequent analysis. The study ensures that unprocessed image samples are not used directly, reducing potential errors in detecting defects on the CBN insert cutting surfaces. The primary steps implemented include image stitching, grayscale transformation, and filtering.

### 2.3. Training Dataset Construction

**Figure 3:**
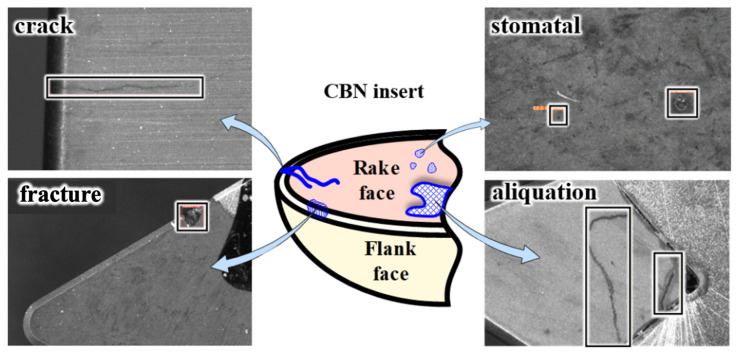
Various types of insert defects.

The various types of defects are described in Figure 3. A comprehensive database capturing a spectrum of CBN insert defects was constructed by photographing defective tools, yielding a corpus of 1320 original images.

The dataset includes four common tool defects: fracture, crack, stomatal, and aliquation. The fracture refers to the damage on the edge of the tool; the crack is a long type defect with a length greater than 0.2 mm and a depth greater than 0.1 mm. The stomatal is the pit, bulge, or flaw on the surface of the tool, which is generally round and spherical; the aliquation refers to the strip defect with a depth of less than 0.1 mm on the tool surface. Each defect captures 330 original images, which are tagged with the help of Makesense, a semi-automatic tool.

These images were labelled with the aid of the semi-automatic tool Makesense.ai and subsequently divided into training and testing sets in an 80–20 split. The final dataset encompasses diverse defect types, such as chipping, cracking, delamination, porosity, and edge chipping, which are prevalent in industrial scenarios. Less common defects, such as non-tangency at round corners, tool head deviations, and size discrepancies, are present but occur less frequently in manufacturing environments. Consequently, these were not included in the present study, allowing for a focused analysis of the most significant and representative defect types encountered during tool testing in production settings.

## 3. System Model Construction

**Figure 4:**
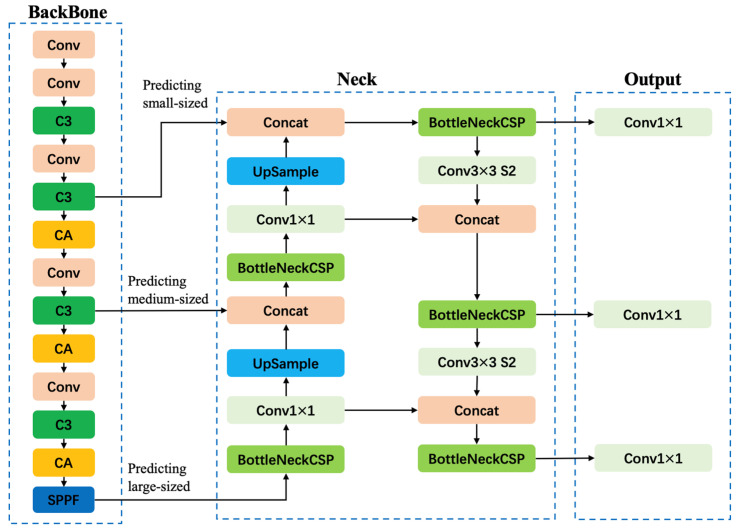
Network structure based on improved YoloV5s.

The YOLO algorithm conceptualizes target detection as a regression problem, efficiently dividing the image into several grids. For each grid cell, it simultaneously predicts bounding boxes and class probabilities. The architecture of the YOLOv5 network is delineated in Figure 4. The YOLOv5s, a variant used in this study, is composed of four essential parts in its network architecture: (i) Input End: At the inception, images are preprocessed to tensors through resizing and normalization to prepare them for neural network compatibility. Data augmentation, including adaptive anchor box calculations and image scaling, is also introduced at this stage to enhance the robustness of the input data. (ii) Feature Extraction: The core of YOLOv5s is designed for feature extraction, consisting of the Backbone, Neck, and Head layers. The backbone is a convolutional neural network that processes images to extract features. In order to better extract features, the CA attention mechanism is introduced. The Neck extends these capabilities by integrating features from different layers of the Backbone to improve the richness of the representation. The Head then applies convolutional and connected layers to the enhanced features from the Neck to predict the position of the target boxes and their respective class probabilities. (iii) Target Detection: The neural network then processes the prepared data, pinpointing the location and classifying each target within the image, employing the features extracted. (iv) Result Output: The final detection results are then outputted, providing a visual display for users and the option to store the data for further analysis or integration into subsequent processing stages. The CA attention mechanism has the advantages of effectively capturing inter channel relationships, being suitable for lightweight networks, and being simple and efficient. The algorithm proposed in this paper adds a CA module at each of the middle, middle, and bottom layers of the backbone network, enabling the model to better extract features of the target of interest and improve its detection performance. The architecture of the improved YOLOv5 network is delineated in Figure 4. Conv stands for Convolution; CA is a coordinate attention mechanism; Concat is the sum of the number of channels in the feature map, while Upsample is the upsampling; SPPF stands for fast spatial pyramid pooling; C3 is the C3 module.

### 3.1. Data Augmentation and Adaptive Anchor Box Processing

**Figure 5:**
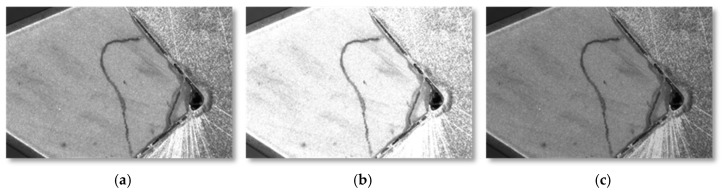

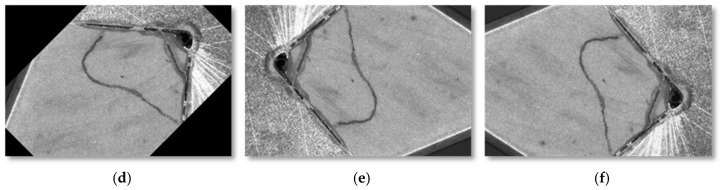
Data offline enhancement. (**a**) Original, (**b**) Brighter, (**c**) Darker, (**d**) Rotate, (**e**) Translation and flip, (**f**) Gaussian noise.

Due to the limited size of the dataset, this paper adopts a method combining offline augmentation and online augmentation. Data augmentation can convert the original dataset into more samples by applying various transformations, thus increasing the size of the dataset. This is achieved through random rotation, random flipping, noise addition, translation, brightness transformation, and adding Gaussian noise to the images. Figure 5 outlines the sequential data augmentation steps applied to the dataset: from the original, subsequent images undergo progressive transformations, including brightness adjustments, rotations, translations with flipping, and the introduction of Gaussian noise to enhance the dataset’s variability and size.

In the process of image acquisition, in order to make the defects more obvious, the defect parts are basically concentrated in the center of the image, thus reducing the problem of loss of defect parts that may be caused by rotation operations.

Data augmentation increases the number of samples in the originally small dataset to around 3000 images, effectively expanding the original dataset by applying multiple transformations, thereby increasing the number of samples.

The study incorporates the Mosaic data augmentation technique, which combines four distinct images to synthesize a new training image. This method enriches the dataset diversity, enhances the model’s robustness, and effectively increases the training dataset size while reducing the likelihood of overfitting. An evaluation involving 3000 images from the training set was conducted to measure the impact of Mosaic augmentation on mean Average Precision (mAP). The results indicate a tripling of the training set size and a five percentage point improvement in mAP subsequent to the augmentation. This increase signifies a considerable boost in the model’s training performance. The expanded dataset enables the model to learn data characteristics better and exhibit improved stability during testing. Although specific outcomes may differ according to the project context, Mosaic data augmentation typically results in a 1.5 times increase in the number of training samples, thereby enhancing the accuracy and robustness of the model, as shown in Table 2.

Anchor boxes are pivotal in target detection, acting as predefined bounding boxes for object prediction within images. Traditional detection algorithms often rely on static anchor boxes, which may not effectively accommodate diverse object dimensions and aspect ratios, leading to decreased prediction accuracy. This study introduces an adaptive anchor box processing technique to reconcile this limitation by aligning anchor boxes more closely with variable object attributes across datasets. The method progresses through several stages: (i) Clustering target boxes from the training dataset by aspect ratio and area, establishing a set of initial cluster centers. (ii) These cluster centers will be adopted as the baseline dimensions for anchor boxes during model training. (iii) Dynamically adjusting the anchor boxes’ dimensions throughout the training, employing learning algorithms to improve their conformity to the dataset’s target objects. (iv) Strategies like non-maximum suppression (NMS) should be implemented to eliminate overlapping predictions and finalize the target detection output. The integration of adaptive anchor box processing has proven beneficial, notably improving both the precision and recall rates of target detection in scenarios with substantial object size and shape variability.

### 3.2. Backbone Structural Layer

Backbone modules are crucial for feature extraction, leveraging high-performance classifier networks to discern common dataset features. In this research, CSPNet, a high-efficiency backbone network, is integrated with YOLOv5 to formulate CSPDarknet53, which serves as our primary backbone network. Furthermore, the Focus structure is implemented as the reference network to amplify computational efficiency without compromising information fidelity. It commences with channel expansion processing, where the Focus module reduces pixel values of the input image and redistributes the width and height dimensions into the channel space, thereby quadrupling the input channel relative to the original RGB channels. Within the CSP network, there are two predominant architectures: CSP1_X and CSP2_X. The CSP1_X is instrumental in the backbone, bifurcating the feature map into two distinct paths. The first path proceeds through convolutional processes, while the second integrates CBL, the ResUnit residual block, and further convolution. These parallel streams are then merged, followed by applying a Batch Normalization (BN) layer and a Leaky ReLU activation function for enhanced feature representation.

Conversely, the CSP2_X architecture operates within the Neck portion of the network. It adopts a similar bifurcation approach, where the first branch is subject to convolution, and the second undergoes dual CBL operations before convolution. The amalgamation of these branches then progresses through a BN layer, concluding with the Mish activation function, providing a robust feature set for subsequent detection tasks.

To determine the position of a target pixel in the source image, the coordinates *src_x_* and *src_y_* denote the pixel’s location in the original image. The specific calculation relationship is defined by the following formula:

(1) $${src}_{x}=\frac{d{es}_{x}\;\;{src}_{w}}{{des}_{w}}$$

(2) $${src}_{y}=\frac{d{es}_{y}\;\;{src}_{h}}{{des}_{h}}$$

In this context, *des_x_* and *des_y_* represent the X and Y coordinates of the corresponding pixel in the target image. Additionally, *src_w_* and *src_h_* specify the width and height of the source image, whereas *des_w_* and *des_h_* refer to the width and height of the target image.

Typically, the values of *src_x_* and *src_y_* calculated using the above formula are floating-point numbers. These floating-point coordinates, derived through inverse transformation, can be expressed as (*i* + *u*, *j* + *v*), where *i* and *j* are the integer components of *src_x_* and *src_y_*, respectively, and *u* and *v* are the fractional components, with *u*, *v* ∈ [0, 1). The pixel value *f*(*i* + *u*, *j* + *v*) at these coordinates can then be estimated based on the values of the four neighboring pixels located at (*i*, *j*), (*i* + 1, *j*), and (*i* + 1, *j* + 1) in the source image.

*f*(*i* + *u*, *j* + *v*) = (1 − *u*)(1 − *v*)*f*(*i*, *j*) + (1 − *u*)*vf* (*i*, *j* + 1) + *u*(1 − *v*)*f*(*i* + 1, *j*) + *uvf* (*i* + 1, *j* + 1) (3)

where *f*(*i*, *j*) represents the pixel value at the source image (*i*, *j*), and so on.

Anchor boxes serve as windows centered on a given point, enabling the detection of multiple overlapping target objects. The initial selection of anchor boxes plays a crucial role in determining both the detection accuracy and the processing speed of the network. The clustering process uses the average degree of overlap, Avg IOU, as the index for target cluster analysis, and is applied to the dataset of CBN inserts. The objective function *f* for Avg IOU in the clustering algorithm can be formulated as follows:

(4) $$f=argmax(\sum_{i=1}^{k}\sum_{j=1}^{n_{k}}I_{IOU}(B,C)/n)$$

*i* denotes the index of a sample, *j* represents the index of the cluster center, and *K* indicates the total number of clusters. IoU(*B*, *C*) represents the intersection-over-union ratio between the bounding box *B* and the cluster center *C*.

### 3.3. Coordinate Attention

In the recognition of defects on tool surfaces, small-sized defects are highly susceptible to the influence of complex backgrounds. Due to the large amount of redundant information generated after convolution, the detection effect of small targets in the image is poor. In order to solve the problem of interference from redundant information, this paper introduces the coordinate attention mechanism (CA). CA attention mechanism (Coordinate Attention) is a lightweight attention mechanism used in mobile networks, aimed at enhancing feature expression ability without increasing computational costs. It decomposes channel attention into two 1D encoding processes, aggregating features vertically and horizontally to capture long-range spatial dependencies and preserve accurate positional information. The CA attention mechanism consists of two parts: coordinate information embedding and coordinate attention generation. Coordinate information embedding aggregates features in both horizontal and vertical directions through 1D pooling operations, generating direction aware feature maps that contain positional information. Then, coordinate attention is generated to combine these directional feature maps and generate attention weights in two directions, which are applied to the horizontal and vertical directions of the input feature map, respectively, to ultimately obtain enhanced output features.

Assuming the size of the input feature map X is C × H × W, use pooling kernels of (H, 1) or (1, W) to perform global pooling encoding on each channel along the horizontal and vertical directions, respectively. Here, *c* is the number of channels and *i*, *j* are the coordinate values. The global pooling encoding formula for each channel is given by Formulas (5) and (6).

(5) $$Z_{c}^{h}\left(h\right)=\frac{1}{W}\sum_{0\leq i\leq W}x_{c}\left(h,i\right)$$

(6) $$z_{c}^{w}\left(w\right)=\frac{1}{H}\sum_{0\leq j\leq H}x_{c}\left(j,w\right)$$

Based on the two features generated above, we further combined the two feature maps, and then used a 1 × 1 convolution to perform a transformation operation on them.

(7) $$f=\delta [F_{1}([Z^{h},Z^{w}])]$$

In the formula, $F_{1}$ is a 1 × 1 convolution transformation function, square brackets represent the combination operation along the spatial dimension, and *δ* is the nonlinear activation function *h*-Swish. We decomposed the intermediate feature map f into two separate tensors $f^{h}\in R^{C/r\times H}\;$ and $f^{w}\in R^{C/r\times W}$, where *r* is the module size reduction rate. We transformed $f^{h}$ and $f^{w}$ into tensors with the same number of channels, and after applying the Sigmoid activation function, obtained the attention weights $g^{h}$ and $g^{w}$ in the height and width directions of the feature map, respectively.

(8) $$g^{h}=\sigma [F_{h}(f^{h})]$$

(9) $$g^{w}=\sigma [F_{w}(f^{w})]$$

Finally, the output feature map $Y\in R^{C\times H\times W}$ of the attention module was obtained, where $F_{h}$, and $F_{w}$ are convolution operations using a kernel size of 1 × 1. [·,·] is a connection operation.

(10) $$y_{c}\left(i,j\right)=x_{c}\left(i,j\right)g_{c}^{h}\left(i\right)g_{c}^{w}\left(j\right)$$

### 3.4. NECK Network

YOLOv5s distinguishes itself within the YOLO series through its specialized Neck network, which incorporates the innovative CSP2 structure. This inclusion markedly bolsters the network’s capacity for feature fusion, enriching the effectiveness of the overall detection mechanism.

Feature Pyramid Networks (FPN) adeptly manage the challenge of detecting objects at multiple scales by constructing a multi-level pyramid that modifies the image resolution at each level. This enables the network to capture and represent features effectively across varying image sizes. Meanwhile, the Pixel Aggregation Network (PAN) leverages a ResNet-18 backbone coupled with a proficient segmentation head that refines feature maps, ensuring heightened efficiency of the network. PAN serves to broaden the receptive field and amplify the feature representation capabilities. In YOLOv5s, a pyramid structure is employed to propagate robust semantic features in a top-down approach, which proves particularly advantageous for delineating larger objects. When integrated with PAN, YOLOv5 further enhances its capability by conveying precise localization details from the bottom layers upwards into the deeper layers of the network, thus clarifying the representation of smaller objects.

### 3.5. Output Head

Within YOLOv5s, the output head acts as the concluding network layer, transforming feature maps into final predictions that include bounding boxes and class probabilities. The head comprises a trio of specialized sub-layers that work in concert. First, a convolutional layer compacts the feature map to align the number of channels with those needed for the bounding box predictions. It performs a max-pooling operation, reducing the channel dimensions to lessen the computational demands. Subsequently, a transposed convolutional layer, often described as the upsampling layer, re-expands the compressed feature map back to its initial scale. Bilinear interpolation is the chosen method for up-sampling within YOLOv5s, and it is chosen for its efficacy in retaining key feature details.

The concluding phase involves the detection layer, which transforms the feature map into an array of bounding boxes and their associated class probabilities. It utilizes activation functions like the sigmoid to ensure accurate predictions. It calculates the positional offsets and dimensions of each bounding box. It assigns class probabilities, bounding the box coordinates within the image frame and the probabilities within a zero-to-one interval. The YOLOv5s head layer seamlessly translates feature maps into actionable predictions via convolution, upsampling, and detection layers. This innovative architecture enables direct processing across the entire image field, circumventing the need for pre-selecting regions or manually identifying object features.

### 3.6. Model Construction Based on Multi-Granularity

**Figure 6:**
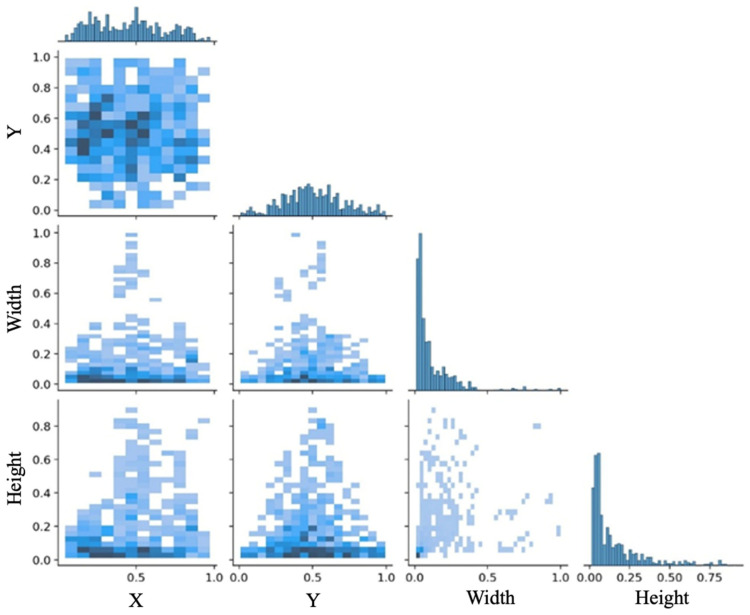
Labels_correlogram graph.

The defect detection process revealed that the size of the defects substantially influences the identification results. Figure 6 presents the distribution of defect center poses. Where X represents the normalized value of the horizontal axis of the anchor box center, Y represents the normalized value of the vertical axis of the anchor box center, Height represents the normalized value of the anchor box height, and Width represents the normalized value of the anchor box width. It can be seen from Figure 6 that the distribution of anchor boxes is not uniform. The model’s performance varied with defect dimensions. A notable issue was the model’s tendency to overlook minor defects, such as bubbles, while readily identifying more significant defects, such as cracks. Conversely, when focusing on more minor defects, the model occasionally misinterpreted more significant defects as continuous white noise, leading to inaccurate detections. These observations underscore the need for further refinement of the model to enhance its sensitivity and accuracy across a range of defect sizes.

This paper introduces a multi-step fine-grained defect detection algorithm leveraging the YOLOv5s framework to circumvent the challenges identified in defect size discrimination. Differentiated models were deployed for various defect sizes. Table 3 shows the comparison of average accuracy between YOLOv5s and the multi-step fine-grained model after 300 rounds of training for defects of different sizes on the same training set. The input for YOLOv5s was a standard 768 × 512 image, while the input for the multi-step fine-grained model was a 6048 × 4096 pixel image and a 768 × 512 pixel image.

This article introduces a multi-step fine-grained defect detection algorithm that utilizes an improved YOLOv5s framework to avoid the challenge of defect size recognition. In response to the problem of difficult optimization of anchor box size in the detection model, differentiated models were deployed according to different defect sizes. For small defects such as stomatal, the input image was enlarged four times proportionally by pixels (i.e., the anchor box is enlarged four times proportionally); on the contrary, for larger defects such as aliquation, cracks, and fracture, the input image was scaled proportionally to 768 × 512 pixels (i.e., the large anchor box was scaled 16 times proportionally). The test outputs were analyzed using DBSCAN clustering, represented by a four-tuple (x, y, w, h). Non-Maximum Suppression (NMS) was employed to merge overlapping detections based on the Intersection over Union (IoU) metric to refine the results. Defects with confidence scores below the established threshold were discarded, with the rest constituting the finalized detection outcomes. The proposed multi-step fine-grained model further im-proves the accuracy of CNB tool defect identification.

## 4. Experimental Validation

### 4.1. Experimental Software Environment

The experimental framework for this study was meticulously constructed on a Windows 10 operating system. The OpenCV 3.4.8 module package powered the core computational processes. To facilitate camera integration and image acquisition, the Multiple Cameras and MvImport toolkits were installed from the MVS development package (SDK: V4.1.0.3). The coding and compilation environments utilized were PyCharm and Anaconda 5.0.0, respectively, with an emphasis on compiling deep neural network (DNN) modules compatible with deep learning frameworks such as Darknet and PyTorch. Furthermore, GPU acceleration was achieved via OpenCL modules, enhancing the efficiency and speed of the experimental computations.

### 4.2. Analysis of Experimental Results

An advanced iteration of the multi-grained YOLOv5s model underwent a rigorous training regimen using a designated dataset. The training protocol was carefully designed to optimize the model’s performance, with the Adam optimizer enhancing the training effectiveness and the cross-entropy loss function, ensuring rapid convergence. Initial conditions were set with a learning rate of 0.0015, a batch size 64, and a comprehensive 300 training epochs to facilitate deep learning. Numerous training iterations and precise parameter calibrations were performed to fine-tune the model’s capabilities to determine the most practical combination of model parameters and hyperparameters.

In addition, the precision (P), recall (R), and mean average precision (mAP) were selected as key evaluation metrics. The precision indicates the ratio of accurately detected objects to the total number of detected objects, while recall represents the ratio of accurately detected objects to the total number of relevant objects in the validation set. Among these metrics, the mAP serves as the most critical indicator in object detection tasks, as it reflects the overall detection performance, averaging AP values of all categories, that is, mAP, as shown in Equation (11).

(11) $$mAP=\frac{1}{n}\sum_{i=1}^{n}\int_{0}^{1}P_{smooth}\left(r\right)dr$$

where *r* is the recall rate, *P_smooth_* is the smoothed P-R curve equation, and *n* is the number of categories.

**Figure 7:**
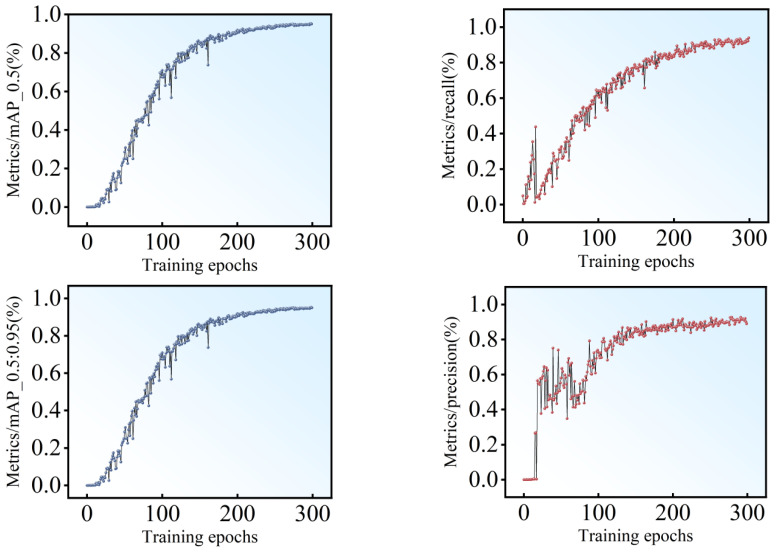
Performance of the mean average precision.

Figure 7 shows the training accuracy of the model. The left column is the mean average precision when the IoU are 0.5 (mAP 0.5) and 0.5 to 0.95 (mAP 0.5:0.95). Whether the mAP 0.5 or mAP 0.5: 0.95, the accuracy of the constructed model approaches 90%, which greatly improves the average detection accuracy. Simultaneously, as the epochs increase, the detection performance of the model exhibit an upward trend as seen from the right column.

**Figure 8:**
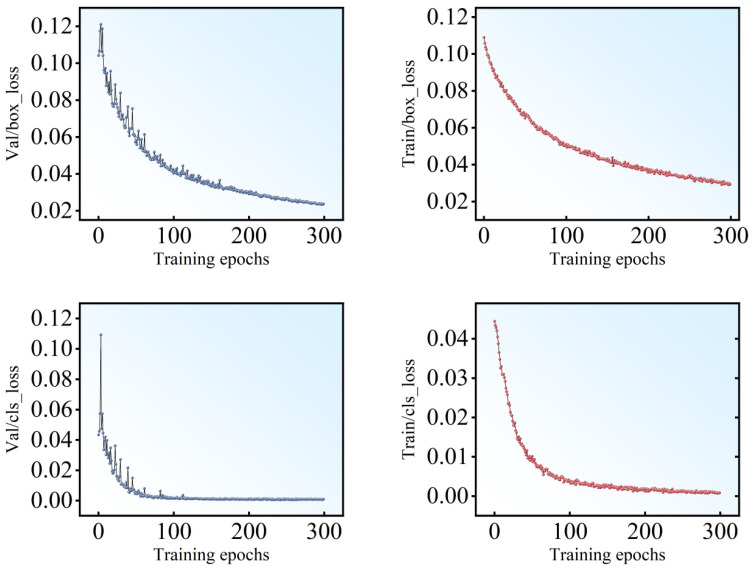

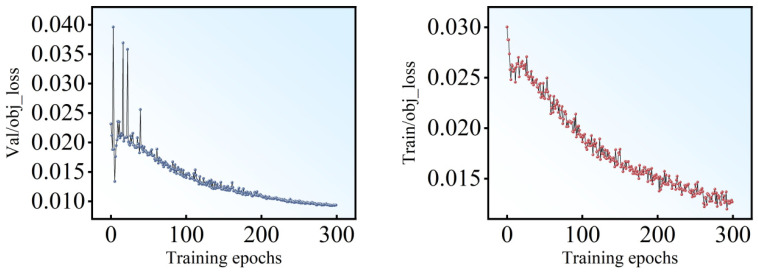
Loss map of YOLOv5s with 300 epochs.

Loss plays an important role in the training process, which reflects the relationship between the true value and the predicted value. The smaller the loss is, the closer prediction value is to the true value, and the better performance of the model. The meticulous training journey and parameter optimization efforts of the network are detailed and visualized in Figure 8. Upon the completion of each training epoch, the model’s performance was rigorously assessed against the validation set. This evaluation encompassed a suite of metrics: bounding box loss (box_loss), mean value of objectiveness loss (obj_loss), classification loss (cls_loss). The localization loss quantified the deviation of the predicted bounding boxes from the ground truth, employing the Mean Squared Error for computation. The confidence loss evaluated the network’s certainty in its predictions, whereas the classification loss, determined by the Cross-Entropy Loss, gauged the difference between the predicted and actual categories. Together, these metrics provided a comprehensive view of the model’s accuracy and predictive abilities at each stage of training. As illustrated in Figure 8, the box_loss, obj_loss and cls_loss for both the training and validation sets exhibit a downward trend, eventually reaching a stable state and converging to a lower loss. For the three index in the validation set, the utilized model is 0.022, 0.001, and 0.009, respectively, at 300 epochs.

**Figure 9:**
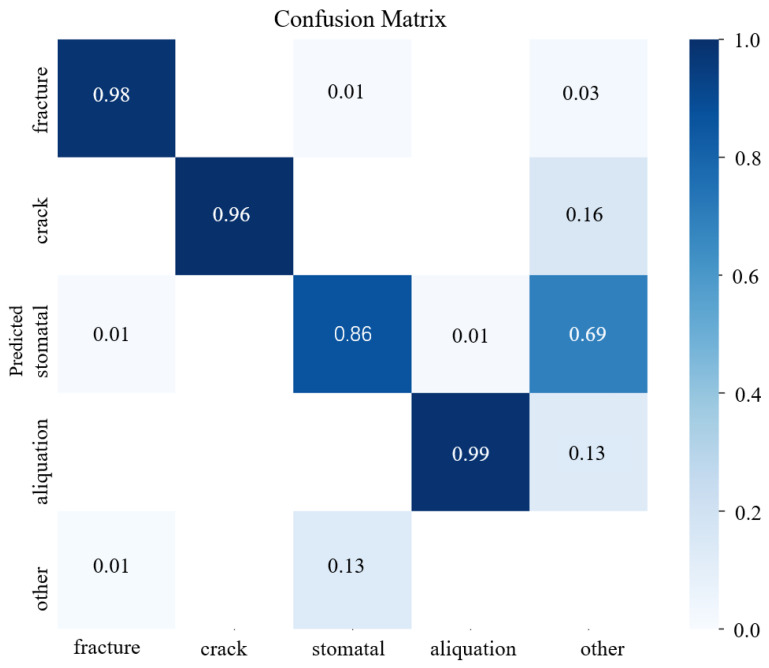
Confusion matrix of defect classification.

The confusion matrix is a table used to evaluate a classification model’s performance, showing the model’s classification accuracy across different categories. The rows of the confusion matrix represent the true labels, and the columns represent the prediction labels. Normalization is used to eliminate the effects and get a clearer picture of the model’s performance in each category, independent of the number of samples for the category. When normalize is True, the values in the confusion matrix are normalized to sample percentages for each class, i.e., the sum of each row becomes 1. The confusion matrix for the major defect class of the tool is shown in Figure 9.

**Figure 10:**
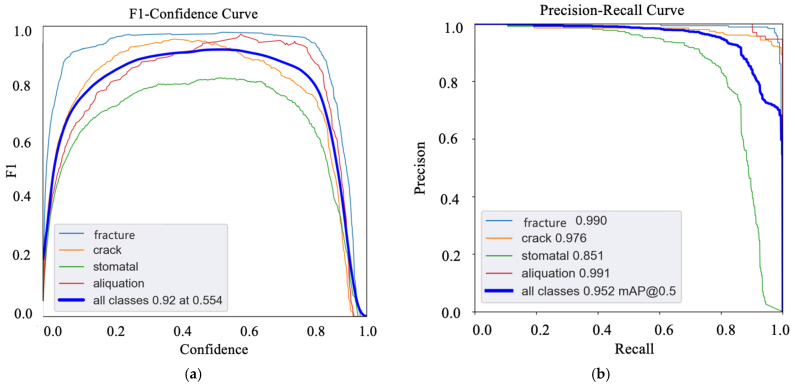
Performance evaluation of the model. (**a**) Recall rate of defects, (**b**) Confidence of defects.

The testing phase focused on evaluating the model’s performance against the designated test set. The model’s effectiveness was quantified using key metrics, including detection accuracy, recall rate, and F1-Score, which are visually represented in Figure 10. On the test set, the model achieved an impressive detection accuracy of 95% and a recall rate of 90%. Furthermore, the model registered a mean Average Precision (mAP) of 0.92 and a mean Intersection over Union (mIoU) of 0.85, attesting to its high accuracy in both detection and localization. A comparative analysis was conducted against renowned object detection algorithms such as Faster R-CNN, SSD, and RetinaNet to corroborate the model’s effectiveness. The empirical results demonstrate that the proposed machine vision-based method for detecting defects in CBN inserts significantly surpasses these algorithms in terms of accuracy and speed. The developed CBN inserts surface defect detection system, underpinned by the machine vision technology discussed in this paper, demonstrates its capability to precisely identify a range of cutting tool defects. The system exhibits significant practical utility and potential for future applications.

### 4.3. Automated Visual Inspection Results

**Figure 11:**
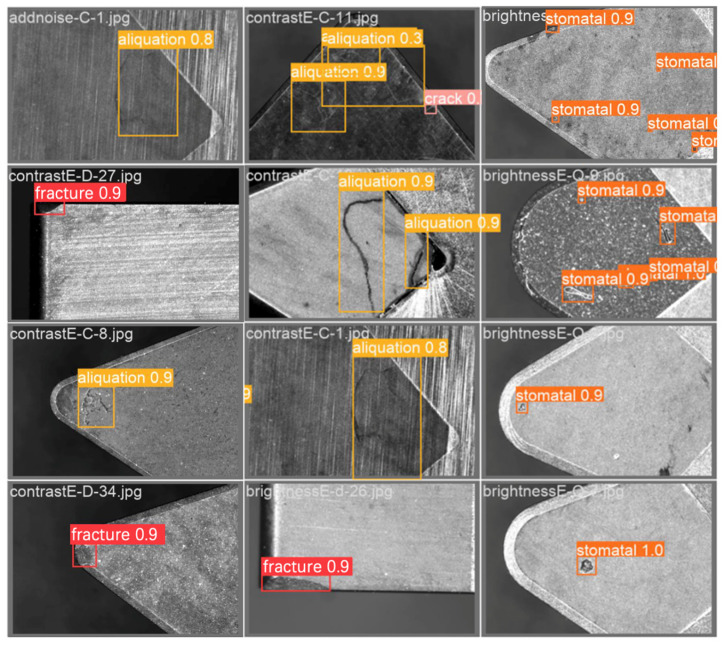
The test results on the validation set.

The built platform of automated visual inspection was used to debug the corresponding parameters for experimental verification. The confidence level was adjusted to 0.3, with 12 images forming a single batch. Then, a batch was randomly selected to be tested on the validation set. The outcomes of these tests are elucidated in Figure 11. The experimental results indicate that all the defects in the cutting tool can be accurately identified, with the confidence level of most defects being greater than or equal to 0.8. The test results fully demonstrate that the trained model can effectively identify CBN cutting tool defects and has specific practical value in industrial inspection scenarios.

**Figure 12:**
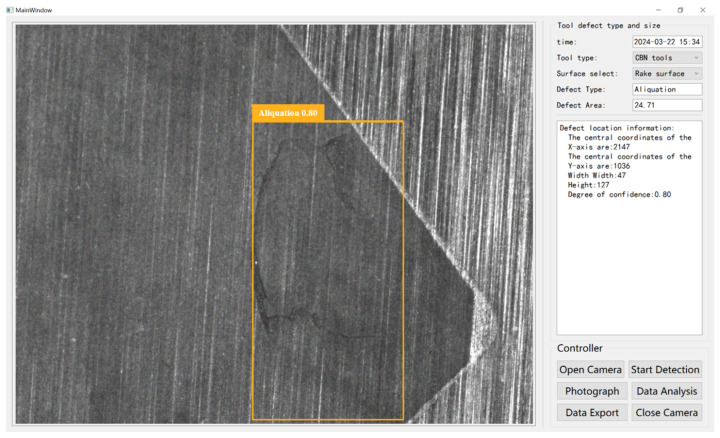
The result of running on UI interface.

The program utilized PyQt 5.15.1 to operate the industrial camera and PyDesigner 5.15.1 to craft the user interface, which was then converted to ‘Ui.py’ for program integration. Given the host computer’s limited capacity for real-time camera display, an adaptive display method was employed to rectify incomplete image results. Figure 12 illustrates the integration of the UI interface with the optical platform, enabling real-time visualization and defect detection in images. The system not only displays but also analyzes image data, pinpointing defect location, size, and area. Moreover, it exports detailed defect information—including type, anchor box coordinates, size, area, perimeter, and confidence level—to a CSV file, while segmented defect images are saved in JPG format.

## 5. Discussion and Conclusions

In the mass production of CBN inserts, it is inevitable that surface defects such as stomatal, segregation, cracks, and fracture, will manifest, markedly diminishing product quality. Traditional manual inspection, the predominant method currently in use, is labor-intensive and prone to human error. Addressing this gap, this paper introduces a visual inspection-based defect detection system for the intelligent analysis of CBN inserts. The following concluding remarks can be drawn.

1. The research presented herein underscores that defect size plays a critical role in recognition accuracy. For defects larger than 25 × 25 pixels, such as delamination and cracks, the model’s accuracy exceeds 95%. In contrast, for smaller defects like stomatal, which are often invisible to the human eye yet critical due to their impact during high-stress tool operation, accuracy falls below 85%. The multi-granularity recognition model developed herein demonstrates appreciable accuracy for detecting defects across sizes, highlighting its potential utility in industrial manufacturing and application processes.
2. The algorithm enhances model robustness and accuracy through data augmentation, adaptive anchor box processing, and adaptive image scaling at the input level. The Backbone structure is optimized with the integration of Focus, CSP, and SPP technologies, enhancing the network’s feature extraction capacity. The Neck network and output Head utilize multi-layer feature fusion to elevate detection precision further. The proposed multi-step fine-grained model for defects of different sizes further improves the recognition accuracy. Experimental results show that compared with the YOLOv5s model, the multi-step fine-grained model has an improved recognition accuracy of 4.7% for small defects and 1.2% for large defects.
3. Experimental results evidence supports the YOLOv5s model’s strong performance in identifying surface defects of CBN insert cutting tools. The model achieves high accuracy rates, with 98.4% for fracture, 95.7% for delamination, 97.6% for cracks, and 85.1% for bubbles. Notably, the test frequency on the local CPU platform deployed and reasoned with ONNX was 137.2 FPS, far more than the 4 FPS required to comply with automatic visual inspection in the factory setup.

In summary, the model presented in this study not only meets but also anticipates the demands of automated inspection systems. The findings and methodologies can serve as a foundation for further model enhancements and adaptations to support and advance industrial production workflows.

## 6. Future Work

Future work will focus on intelligent management for insert life leveraging the developed system and enriching the established database.

## Figures and Tables

**Table 1:** THE camera and computer specifications.

Parameter	Value
Camera	Hikvision, MV-CS060-10GM
Pixel size	2.4 µm × 2.4 µm
Resolution ratio Exposure time	3072(H) × 2048(V)
Field of view	3.69 mm × 2.46 mm
Illumination	Annular LED white light
Lens	1/1.8 inches
Software for processing	Anconda3.8 python3.9
Computer	Windows10 GeForce GTX 285

**Table 2:** Comparison of Mosaic data enhancement effects.

Data Augmentation Method	Training Set Size	mAP Improvement
Without data augmentation	3000	0.82
Enhance with Mosaic data	12,000	0.87

**Table 3:** Comparison of accuracy of different models.

Model	Size (Pixels)	Acc (Aliquation)	Acc (Stomatal)
YOLOv5s	768 × 512	0.85	0.81
Multi-step fine-grained model	6048 × 4096, 768 × 512	0.96	0.94

## Data Availability

Data are contained within the article.
